# Usefulness of Collaborative Work in the Evaluation of Prostate Cancer from MRI

**DOI:** 10.3390/clinpract12030040

**Published:** 2022-05-20

**Authors:** Christian Mata, Paul Walker, Arnau Oliver, Joan Martí, Alain Lalande

**Affiliations:** 1 Pediatric Computational Imaging Research Group, Hospital Sant Joan de Déu, 08950 Esplugues de Llobregat, Spain; 2 Research Centre for Biomedical Engineering (CREB), Barcelona East School of Engineering, Universitat Politècnica de Catalunya, 08019 Barcelona, Spain; 3 ImViA Laboratory, Université de Bourgogne Franche-Comté, 64 Rue de Sully, 21000 Dijon, France; pwalker@u-bourgogne.fr (P.W.); alain.lalande@u-bourgogne.fr (A.L.); 4 Institute of Computer Vision and Robotics, University of Girona, Campus Montilivi, Ed. P-IV, 17003 Girona, Spain; arnau.oliver@udg.edu (A.O.); joan.marti@udg.edu (J.M.)

**Keywords:** prostate cancer, collaborative work, MRI, manual annotations

## Abstract

The aim of this study is to show the usefulness of collaborative work in the evaluation of prostate cancer from T2-weighted MRI using a dedicated software tool. The variability of annotations on images of the prostate gland (central and peripheral zones as well as tumour) by two independent experts was firstly evaluated, and secondly compared with a consensus between these two experts. Using a prostate MRI database, experts drew regions of interest (ROIs) corresponding to healthy prostate (peripheral and central zones) and cancer. One of the experts then drew the ROI with knowledge of the other expert’s ROI. The surface area of each ROI was used to measure the Hausdorff distance and the Dice coefficient was measured from the respective contours. They were evaluated between the different experiments, taking the annotations of the second expert as the reference. The results showed that the significant differences between the two experts disappeared with collaborative work. To conclude, this study shows that collaborative work with a dedicated tool allows consensus between expertise in the evaluation of prostate cancer from T2-weighted MRI.

## 1. Introduction

Prostate cancer (PCa) has shown a substantial decline in the past 5 years, between 5% and 16%, but it continues to be the most common cancer among men [1]. Our study is focused on the analysis of MR images acquired in the context of PCa. Indeed, it remains one of the most commonly diagnosed solid tumour types in men and an MRI is one of the most efficient imaging modalities used to detect PCa early in its course [2]. Collaborative work is a growing field of work, and understanding how groups learn effectively is critical [3]. The role of radiology in the diagnostic process, focusing on key concepts of information and communication, as well as key interpersonal interactions of teamwork, collaboration, and collegiality, all based on trust, have been explored in previous works [4].

The annotation of medical images is subject to an inherent inter-variability between experts, and in some cases, there are also significant differences between the annotations of the same expert (intra-variability) [5]. This difficulty in annotating the medical findings is due to different reasons, including the quality of the images themselves (difficult to understand, low resolution, and/or subtle changes, etc.), the expert who is performing the annotations (experience, tiredness, etc.), and the working conditions (monitor, annotating device, illuminance, etc.). It is commonly accepted that one way to reduce the variabilities is by overlapping the annotations performed by different experts and perform blindly with respect to the other experts. In this paper, we show that, by using a collaborative approach, the variabilities between experts can be minimized considerably.

Among the techniques used to detect PCa, MRI allows the non-invasive analysis of the anatomy and the metabolism in the entire prostate gland. MRI has been established as the best imaging modality for the detection, localization, and staging of PCa on account of its high resolution, excellent spontaneous contrast of soft tissues, and the possibilities of multi-planar and multi-parameter scanning [6]. Previous works about the manual annotation and evaluation analyses were presented by Meyer et al. [7]. In recent literature, a large-scale annotation of biomedical data and expert label synthesis were presented by Chen et al. [8]. In this work, a state-of-the-art in imaging, treatment, and computer-assisted intervention in the field of endovascular intervention is discussed. More specific works are also focused on the volumetric measurement of hepatic tumours by studying the accuracy of manual contouring using computed tomography (CT) [9]. In the same perspective, Bø et al. [10] investigated the intra-observer variability in low-grade glioma (LGG) segmentation for a radiologist without prior segmentation experience. Indeed, the usefulness of collaborative work between radiologists and medical experts is gaining importance.

The principal problem encountered in the diagnosis of prostate cancer is the localization of a ROI containing tumour tissue. Normally, experts use different tools to establish the diagnoses using different software and make many annotations in different files [11]. This is not a practical solution to managing abundant medical data. The use of a specific dedicated tool allows experts to analyze the prostate gland on T2-weighted imaging (T2WI), diffusion weighted imaging (DWI), perfusion based on dynamic contrast enhancement (DCE), and magnetic resonance spectroscopy (MRS) panels within the same application [12]. In this sense, one of the most evident advantages of this kind of tool is that it allows simultaneous analysis of the prostate using different image modalities and, if available, MRS. More recent works confirm that this interaction between MRI techniques facilitates, for radiologists and medical experts, and the evaluation of the prostate using the PI-RADS v2 classification [13].

In this paper, we compare the delimitation of different ROIs on images of the prostate gland between an independent evaluation, using collaborative work of different experts. The idea behind this study is to show that collaborative work allows a real consensus between experts and potentially decreases variabilities in their evaluation. To this end, the evaluation procedure, evaluation parameters, and the data analysis discussion of the obtained results are presented in this work.

## 2. Materials and Methods

### 2.1. Database

A database containing MRI of both healthy and tumour-bearing prostates was used. The examinations used in our study contained three-dimensional T2-weighted fast spin-echo (TR/TE/ETL: 3600 ms/143 ms/109, slice thickness: 1.25 mm) images acquired with in-plane sub-millimetric pixel resolution in an oblique axial plane. From the 10 patient datasets included in our study, each dataset was composed of 64 slices. In all, 238 annotations were manually delineated by two radiologists.

All the datasets and ground truth data were provided from the Medical Imaging department of the University Hospital of Dijon (France). We report results derived from the analysis of a small but select sample dataset, which was within reach of only a few clinical cases provided by Hospital of Dijon (France). The included cases fulfilled very specific criteria and may be considered as main impacts, in terms of incidence according to the ground truth. The multi-modal MR approach we employed ensured the precise characterization of each case. To the best of our knowledge, this work is the first to analyze such a sample in detail. For this reason, this study is the first step toward obtaining a reliable ground-truth, without expert variabilities, in which automatic algorithms could be robustly compared.

The institutional committee on human research approved the study, with a waiver for the requirement for written consent, because MRI and MRSI were included in the workup procedure for all patients referred for brachytherapy or radiotherapy. As the data were retrospectively collected and untraceable, an ethical approval number, such as an IRB study number, was not needed according to French law. The annotations were performed using our own in-house developed tool [12].

### 2.2. ROIs of Prostate Anatomy

**Figure 1:**
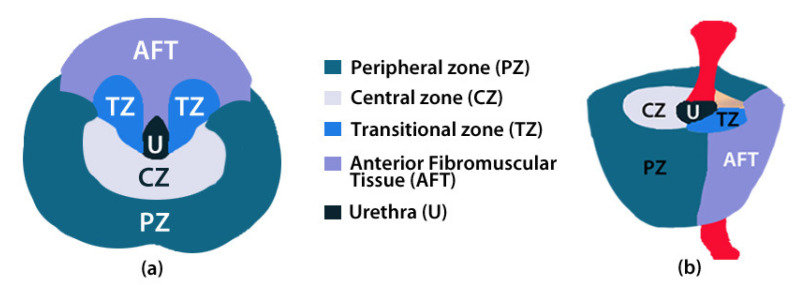
Anatomy of the prostate in (**a**) transversal and (**b**) sagittal planes [11].

The prostate is composed of a peripheral zone (PZ), a central zone (CZ), a transitional zone (TZ), and anterior fibromuscular tissue (AFT) (Figure 1). Most cancer lesions occur in the peripheral zone of the gland, some occur in the TZ whilst very few arise in the CZ. A detailed description of the influence of the prevalent factor risks according the prostate zone is given in [14].

**Figure 2:**
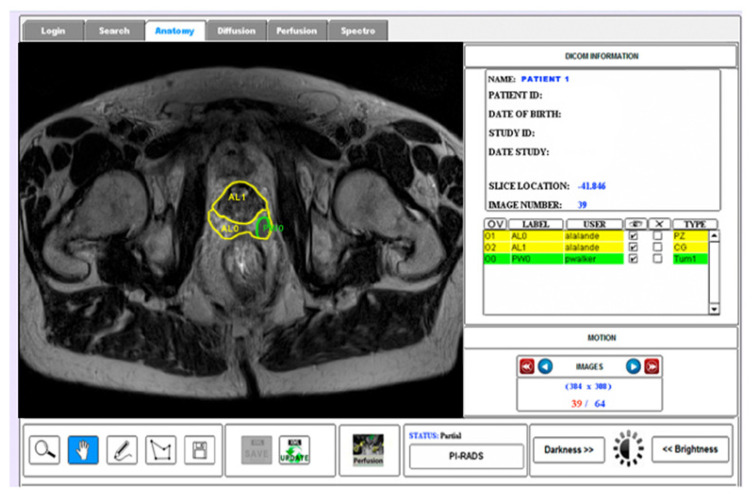
Example of the visualization of the anatomy (T2WI) modality with the corresponding annotations of a prostate gland [12].

Manual drawing of the different ROIs of the prostate according to the prostate anatomic regions and tumour lesion was performed on T2WI. Indeed, due to the high volume of information present in the anatomic images, the purpose of the present study was to evaluate the variability between experts concerning medical findings in prostate gland regions using T2WI (Figure 2). The T2WI modality was chosen because it provides the best depiction of the prostate’s zonal anatomy.

### 2.3. Evaluation Procedure

Experts drew ROIs on the prostate zones corresponding to PZ, CZ, and Tum. In our study, TZ was considered a part of the CZ because it was difficult to distinguish the two zones on the T2WI images. The T2WI sequence did, however, provide excellent contrast between PZ and CZ tissues [15].

**Figure 3:**
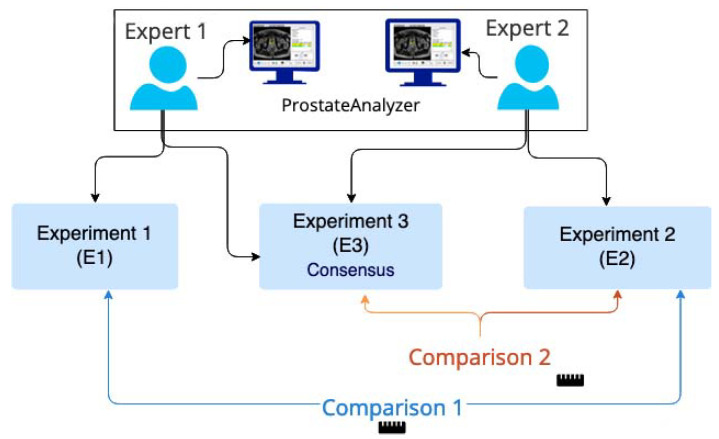
Flow diagram used for the evaluation procedure.

Figure 3 shows a flow diagram of the evaluation procedure. More precisely, the first experiment E1 was composed of the evaluation provided by the first expert. It consisted of drawing ROIs of the prostate gland zones on as many different slices as necessary. For each ROI, the surface area was calculated and then the volume of each zone was estimated from the surface area multiplied by the slice thickness. Similarly, a second experiment E2 was carried out independently by a second expert in the same manner as E1. Finally, the first expert repeated the processing step with a knowledge of the evaluation performed by the second expert (experiment E3). The two experts had more than 10 years of experience in prostate MRI and although formally ranking the experts was not thought to be necessary, we chose to prioritize the second expert (results from E2) given his more regular acquaintance with prostate MRI on a weekly basis. For this reason, E2 was considered as the experiment of reference according to the provided ground truth. This means that the comparison procedure to evaluate the influence of collaborative work was performed in two steps: firstly, E1 vs. E2 and then E3 vs. E2. Only the consensus between experts was considered. A minimum time interval between E1 and E3 was imposed to prevent the expert from using prior knowledge of his previous tracing. This interval was greater than one month in our study [16].

**Figure 4:**
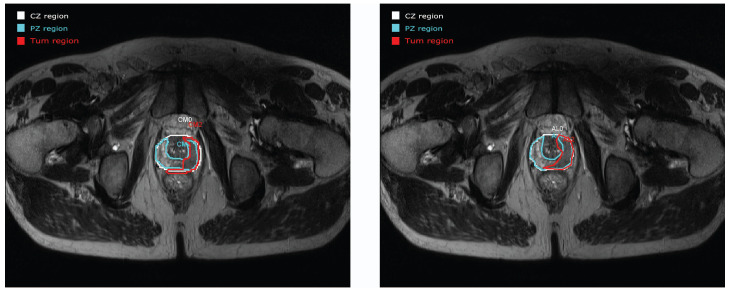
Example of the prostate gland processing from E1 (**left**) and from E2 (**right**). Note the similitude between the two cases.

Figure 4a depicts an example of the prostate gland analysis with a manual drawing of the CZ (in white), PZ (in blue), and tumour area (in red), corresponding to anatomic areas used to make our evaluation. Firstly, we asked the two experts to draw the ROIs independently on several MR examinations. Secondly, one expert redrew his ROIs with knowledge of the evaluation of the other expert. Differences in the contour tracing, such as seen in the volume calculations of the different structures, were compared in order to verify whether a significant improvement of consensus in the results with collaborative work had been observed.

### 2.4. Evaluation Parameters

The correlation coefficient, the regression analysis, and the Bland–Altman [17,18] plot were used to compare the surfaces obtained from E2 with those obtained from E1 and E3, respectively. It is important to notice that the comparison between E3 and E1 was not performed because the evaluation must take into account the E2 as the reference. A linear correlation estimation between E1 and E2, and then E3 and E2, was performed using a two-sample *t*-test [19]. A p-value of less than 0.05 was considered as a statistically significant difference. Moreover, the contours obtained from experiment E2 were compared with the ones obtained with E1 and then with E3.

Firstly, an edge-based approach using the Hausdorff distance [20] in order to do this comparison was used. Hausdorff measures how far two subsets of a metric space are from each other. The definition—let *X* and *Y* be two non-empty subsets of a metric space (M,d). We define their distance by Equation (1) [21].

(1) $$\begin{array}{c} d_{H}\left(X,Y\right)=max\left\{\begin{array}{c} \underset{x\epsilon X}{\sup}d\left(x,Y\right),\underset{y\epsilon Y}{\sup}d\left(X,y\right) \end{array}\right\} \end{array}$$

where sup represents the supremum. Inf corresponding to the infimum quantifies the distance from a point aϵX to the subset B⊆X represented in Equation (2).

(2) $$\begin{array}{c} d\left(a,b\right)=\underset{b\epsilon B}{\inf}d\left(a,b\right) \end{array}$$

Secondly, a region-based approach with the Dice index, also known as the Sørensen–Dice index, were considered [22]. It is a statistical tool that measures the similarity between two sets of data. The equation for this concept is represented in Equation (3) [23].

(3) $$\begin{array}{c} 2*|X\cap Y|/(|X|+|Y|) \end{array}$$

where *X* and *Y* are two sets, a set with vertical bars on either side refers to the cardinality of the set, i.e., the number of elements in that set, e.g., |X| means the number of elements in set *X*, and ∩ is used to represent the intersection of two sets, and means the elements that are common to both sets.

The mean and the standard deviation of each parameter for the whole data set were calculated. Again, a two-sample *t*-test was used to verify if there were any significant differences between the calculation of these parameters. Finally, for each zone, the number of cases in which one expert considered it as being present on one image (i.e., drew the corresponding area) but not so for the other expert, were counted and presented as a percentage of the total number of processed slices by the second expert.

## 3. Results

**Figure 5:**
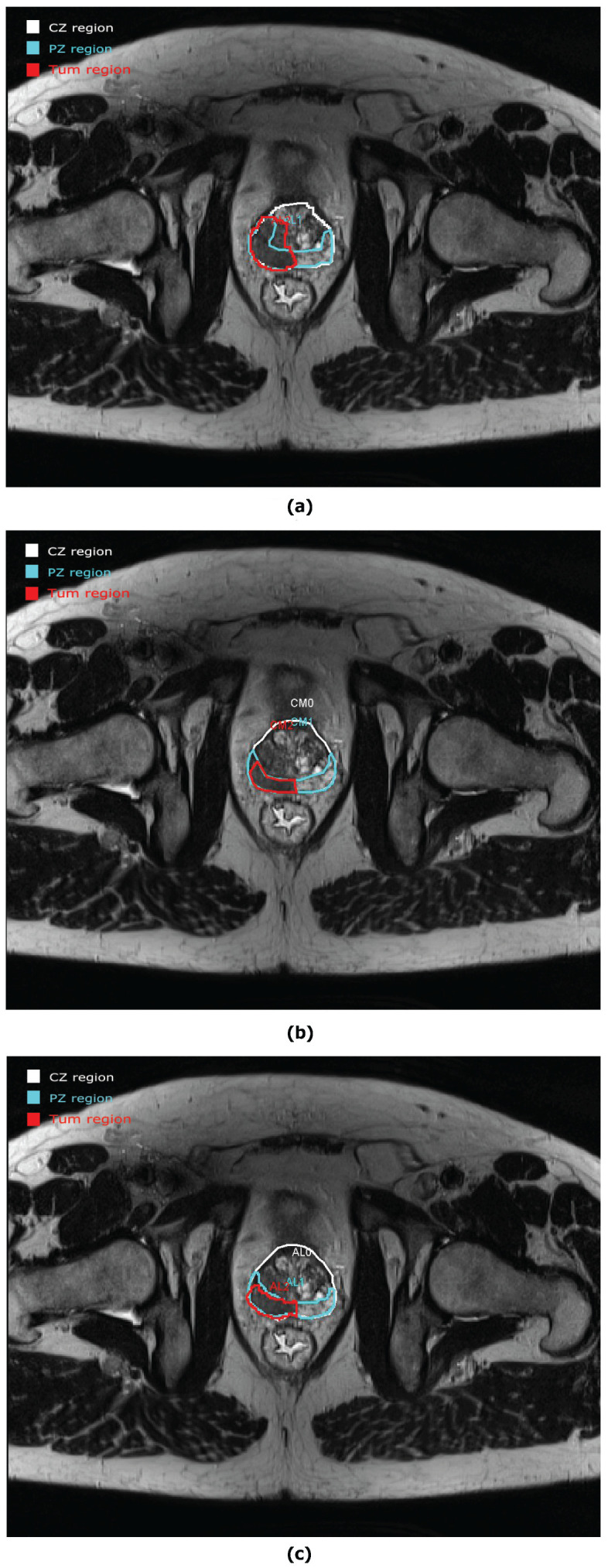
Example of a prostate study evaluation from (**a**) E1 and (**b**) E2 with a discordance between both drawings for the tumour area. (**c**) New evaluation of the prostate study from E3 with a good agreement for the tumour area between E3 and E2.

Two examples of PCa analysis are presented in Figure 4 and Figure 5. The left image in Figure 4 corresponds to the drawing by the first expert E1 and in the right image by the second expert E2. Three ROIs were drawn in images corresponding to CZ (white area), PZ (blue area), and tumour (red area). When visually comparing the two drawings, a very good concordance between CZ and PZ areas can be observed. Concerning the area corresponding to the tumour, a small deviation is seen but contours can be considered as being relativity close between the two experiments.

However, not all the prostate studies were evaluated with such good concordance between experiments. An example of discordance is seen in Figure 5. CZ and PZ have a good approximation between E1 and E2 but an important discordance is seen for the tumour area. A new evaluation was carried out for E3 in Figure 5c. In this example, we can see the real advantage of collaborative work. After collaboration, the tumour areas are approximately the same.

### 3.1. Anatomic Parameters

From Table 1, it is clear to see that the number of cases where either expert does not include a particular zone reduces significantly after collaboration. Indeed, for CZ, this percentage is equal to 12% between E1 vs. E2 and 3% between E3 vs. E2. For PZ, this percentage is equal to 9% between E1 vs. E2 and 3% between E3 vs. E2. Finally, for the tumour, it is 13% between E1 vs. E2 and 0% between E3 vs. E2.

The correlation coefficient (r), regression line, Bland–Altman, and two-sample *t*-test calculated for the area of the three prostate gland zones are depicted in Table 2 and Table 3. In general, the results are improved between E3 vs. E2 compared with E1 vs. E2. More precisely, the correlation coefficient of the area is improved for E3 vs. E2 whatever the considered area.

The Bland–Altman test shows a better agreement between E3 vs. E2 than E1 vs. E2. Incidentally, a Bland–Altman test has also been calculated for the volume evaluation. According to the two-sample *t*-test, there is no significant difference between E3 vs. E2 whatever the considered area, while there are always significant differences in the results between E1 vs. E2. For CZ, it is $40\pm 17\;{\mathrm{mm}}^{2}$ between E1 vs. E2 and $-0.9\pm 3\;{\mathrm{mm}}^{2}$ between E3 vs. E2. For PZ, it is $20\pm 13\;{\mathrm{mm}}^{2}$ between E1 vs. E2 and $3\pm 12\;{\mathrm{mm}}^{2}$ between E3 vs. E2. Finally for tumour, it is $7\pm 6\;{\mathrm{mm}}^{2}$ between E1 vs. E2 and $0.4\pm 0.9\;{\mathrm{mm}}^{2}$ between E3 vs E2.

**Figure 6:**
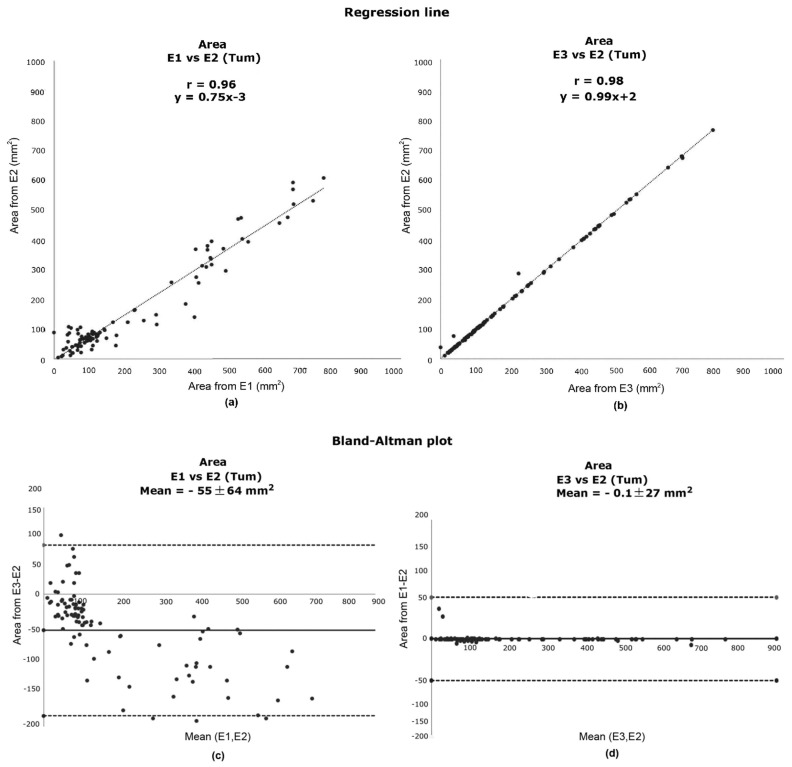
Comparison of tumour surface areas: Regression analysis obtained for (**a**) E1 vs. E2 and (**b**) E2 vs. E3 and the corresponding Bland–Altman plots obtained for (**c**) E1 vs. E2 and (**d**) E2 vs. E3.

Figure 6a,b detail the linear regression analysis for the evaluation of the tumour area. The tumour area has been chosen due to its importance and because this area is more difficult to analyze and provides more variations among experts. When comparing the two obtained regression lines, an improvement is noted in Figure 6b with a slope of 0.99 compared with Figure 6a with a slope of 0.75. Figure 6c,d detail the corresponding Bland–Altman plots. In Figure 6d, it can be seen that the mean of the difference between E3 vs. E2 is close to zero, meaning that there is little bias between the two measurements.

### 3.2. Contour Evaluation

The Hausdorff distance and the Dice index between the different annotations are presented in Table 4. Again, between E3 vs. E2, an improvement is observed with respect to the results obtained between E1 vs. E2. The mean Hausdorff distance is reduced in all the cases. In the same way, the analysis of the Dice index is around 0.9 between E3 vs. E2, whatever the area, whereas it is no higher than 0.7 for E1 vs. E2. The differences between E1 vs. E2 and E3 vs. E2 are always significant, whatever the considered parameter.

## 4. Discussion

Currently, the ground-truth is often obtained via evaluations from different experts performing their tasks independently, and only afterwards, their results are objectively (or subjectively) merged. Ghose et al. proposed a set of open problems mainly related to the evaluation procedure [24]. This can be summarised as (1) variabilities in the ground-truth, (2) unavailability of public prostate datasets, and (3) lack of standardised metrics for evaluation.

Although interpersonal interactions are difficult to specify and quantify, they are critical to the effective flow of information, which itself is critical to the diagnostic process as it is increasingly performed within and among professional teams [4]. It is well known that medicine is becoming increasingly specialised, care teams have less time to care for patients, and interprofessional collaboration in healthcare is more important than ever. Based on the fact that collaborative work is difficult and time-consuming, medical tools are needed to facilitate the decision-making process. Indeed, there is no universal tool that can solve all the shortcomings in healthcare decision-making tasks [25,26]. We believe the work presented in this paper presents the roots for designing an adequate process for prostate evaluation. Moreover, the collaborative work presented in this study is the first step for obtaining a reliable ground-truth, without expert variabilities, in which automatic algorithms could be compared.

In our paper, we studied the usefulness of collaborative work, where the ground-truth is obtained by two experts, but with the second expert having prior knowledge of the other expert’s work. Exhaustive evaluations of the medical findings in different regions of the prostate gland from T2WI were performed. We asked two experts to make these drawings independently on several MR examinations, and as a second step, one expert repeated the drawings with the knowledge of the evaluation of the other expert.

The novelty in our study is to evaluate the variability between experts concerning medical findings in prostate gland regions. The localization of a lesion in a specific area is crucial and the segmentation process remains a challenge. The differences observed for the delimitation of the different ROIs between independent evaluation or using collaborative work by different users was studied. These segmentations remain difficult, and their delineations are fundamental for assigning a PI-RADS score. Differences in the obtained results (e.g., such as in the volume calculations) were compared in order to verify whether a significant improvement in consensus had been obtained through collaborative work. The idea behind this study is to show that collaborative work allows a real consensus between experts and potentially decreases variabilities in their evaluation.

A main limitation of this work is the small sample size. However, it was a select sample dataset that was within reach of only a few clinical cases. The included cases fulfilled very specific criteria and may be considered as main impacts in terms of incidence according to the ground truth. For instance, this work is a proof of concept and we think that increasing the data set will not considerably alter the results, nor the conclusion, as there is already a significant difference between the approaches even with this small data set. A potential bias in our study could be the fact that the second expert participated in the consensus (Experiment E3). However, the time interval of greater than one month between E1 and E3 will considerably limit such a bias. Moreover, the lack of histopathology data are also a limitation in studies concerning prostate cancer. However, our patient data set was extracted from a pool of patients destined to receive radiotherapy treatment as opposed to radical prostatectomy. Therefore, in this context, surgery was out of the question. Moreover, it is complicated to obtain from the biopsy accurate knowledge on the spatial distribution of the tumour within the gland because it is a relatively random procedure.

Another novelty of our study was to show that the evaluation of medical examinations with collaborative work drastically reduces the differences between processing; even this result was expected. In particular, significant differences between the two experts virtually disappeared when there was collaborative work. We probably cannot conclude that the diagnosis was improved, but we do observe vastly improved consensus between experts. There may be human errors in the evaluation that could lower the correctness of the results and increasing the number of experts could diminish this bias. This can be done by a tool allowing working online [11]. However, in general, a consensus of two experts still involves an increase in the quality of the diagnosis.

An alternative point of view would be to affirm that the experiment E3 is biased. While it is true that inter-rater agreements for prostate MRI are not outstanding and that we have not presented such a study in our work, it must be emphasised that our paper is focused on how to counter such problems of heterogeneity of response from experts. Indeed, our study shows that from a relatively dispersed set of the initial data, our approach is capable of reducing such inherent differences. An additional experiment could be performed: the second expert could repeat the process knowing what had been done by the first expert. However, in our opinion, this is not necessary because the main objective of this study was to objectively show that collaborative work in current clinical practice can provide a real consensus between experts, even if there is potentially a bias in the evaluation process. We can have two experts at the same time, and then the scenario is a little bit different, because in this case, there is no specific order between the experts. Furthermore, our protocol evaluation is perfectly in line with the recommendations of PI-RADS v2 in that 3D T2-weighted sequences are expected in the place of the 2D version. Moreover, recent articles at 3T have shown that 3D T2 images are equivalent to the 2D T2 image [27].

Although artificial intelligence (AI) shows promise across many aspects of radiology, the use of AI to create differential diagnoses for rare and common diseases has not been demonstrated [28]. Recent advances using deep learning have brought the immense scope of automatic detection and recognition at very high accuracy in prostate cancer. Automated deep learning systems have delivered promising results from histopathological images to accurate grading of PCa. Many studies have shown that deep learning strategies can achieve better outcomes than simpler systems that make use of pathology samples [29]. There are other examples of algorithms based on artificial intelligence and machine learning in PCa that could be an excellent addition to our work [30,31,32]. Finally, considering the difficulties to segment the prostate gland regions, a solution based on AI was proposed by Bardis et al. [33]. The purpose of their study was to build upon these prior efforts by using a larger data set and two parallel neural networks that were specialised in localization and classification for both TZ and PZ segmentation. In general terms, there are benefits of collaboration work in healthcare, such as improving patient care and outcomes, reducing medical errors, and even improving staff relationships and job satisfaction.

## 5. Conclusions

In this paper, the interest of collaborative work in the evaluation of cancer issues from MRI is presented. Even if improved results had been expected, this study shows that the evaluation of medical examinations with knowledge of the work of another expert, drastically reduces the differences between processing. In particular, significant differences between experts become non-significant when there is collaborative work. We cannot conclude that the diagnosis was improved, but only that there is improved consensus between the experts (but in general, this did involve an increase of the quality of the diagnosis). Moreover, an alternative point of view is to affirm that the results from collaborative work are biased. In fact, it is out of the scope of our study because the main objective was to objectively show that collaborative work in current clinical practices can provide a consensus between experts even if there is potentially a bias in the evaluation process.

In conclusion, although collaborative work requires more time, it allows the improvement of the management of patients with prostate cancer by providing consensual diagnosis, in particular in complex cases.

## Figures and Tables

**Table 1:** Total number of cases for each area (CZ, PZ, and tumour) that have not been evaluated by the two experts between E1 and E2, and E2 and E3.

Patient	Processed Slides	CZ		PZ		TUM	
		E1 vs. E2	E3 vs. E2	E1 vs. E2	E3 vs. E2	E1 vs. E2	E3 vs. E2
Patient 1	18	6%	6%	11%	0%	0%	0%
Patient 2	21	10%	10%	10%	10%	0%	0%
Patient 3	25	8%	0%	8%	0%	12%	4%
Patient 4	30	7%	0%	7%	0%	17%	0%
Patient 5	24	13%	0%	13%	8%	13%	0%
Patient 6	17	18%	0%	12%	0%	65%	0%
Patient 7	26	12%	4%	8%	0%	19%	0%
Patient 8	31	19%	10%	3%	6%	0%	0%
Patient 9	21	14%	0%	14%	0%	5%	0%
Patient 10	25	16%	0%	8%	0%	8%	0%

**Table 2:** Analysis of the correlation coefficient (r) and regression line calculated for the areas of different zones (in mm${}^{2}$). E2 is the reference and is compared with E1 and E3.

	r		Regression Line	
	E1 vs. E2	E3 vs. E2	E1 vs. E2	E3 vs. E2
CZ	0.95	0.98	y = 0.9x − 166	y = x − 12
PZ	0.91	0.94	y = 0.9x − 96	y = 0.9x + 21
TUM	0.96	0.98	y = 0.7x − 3	y = x + 3

**Table 3:** Results obtained from the Bland–Altman plot and *t*-test for CZ, PZ, and the tumour (TUM) area calculated (in mm${}^{2}$) found in the prostate gland using the surface as the anatomical parameter. Again, E2 is the reference and is compared with E1 and E3.

	Bland–Altman		*t*-Test	
	E1 vs. E2	E3 vs. E2	E1 vs. E2	E3 vs. E2
CZ	−261.13 ± 168.20	−13.07 ± 118.09	0.01	0.36
PZ	−156.50 ± 95.71	−10.73 ± 84.60	0.01	0.32
TUM	−54.93 ± 64.34	−0.08 ± 27.13	0.02	0.47

**Table 4:** Analyses of Hausdorff distance (in mm) and the Dice index for the CZ, PZ, and tumour area (TUM). A *p* of <0.05 between E1 vs E2 and E3 vs E2 is found in all the cases.

	Hausdorff Distance		Dice Index	
	E1 vs. E2	E3 vs. E2	E1 vs. E2	E3 vs. E2
CZ	8 ± 3	4 ± 1	0.70 ± 0.20	0.90 ± 0.10
PZ	11 ± 5	5 ± 2	0.60 ± 0.20	0.90 ± 0.10
TUM	10 ± 4	8 ± 11	0.70 ± 0.10	0.90 ± 0.10

## Abbreviations

The following abbreviations are used in this manuscript:

PCa	prostate cancer
ICD-O	International Classification of Diseases for Oncology
ICCC	International Classification of Childhood Cancer
EU	European Union
CT	computed tomography
MRI	magnetic resonance imaging
ROI	region of interest
T2WI	T2-weighted imaging
DWI	diffusion weighted imaging
DCE	perfusion based on the dynamic contrast enhancement
MRS	magnetic resonance spectroscopy
PI-RADS	prostate imaging-reporting and data system
LGG	low-grade glioma
CZ	central zone
PZ	peripheral zone
TZ	transition zone
AFT	anterior fibromuscular tissue
Tum	tumour lesion
*E*1	experiment 1
*E*2	experiment 2
*E*3	experiment 3
